# Different significance between intratumoral and peritumoral lymphatic vessel density in gastric cancer: a retrospective study of 123 cases

**DOI:** 10.1186/1471-2407-10-299

**Published:** 2010-06-17

**Authors:** Xiao-Lei Wang, Jian-Ping Fang, Ru-Yong Tang, Xi-Mei Chen

**Affiliations:** 1 Department of Gastroenternology, Institute of Digestive Disease, Tongji Hospital affiliated to Tongji University, Shanghai 200065, PR China; 2 Department of Pathology, Tongji Hospital affiliated to Tongji University, Shanghai 200065, PR China

## Abstract

**Background:**

Patients with gastric cancer in China have worse outcome and poorer prognosis. Tumor-induced lymphangiogenesis plays a crucial role in metastasis and tumor progression. The intratumoral and peritumoral lymphatics were supposed to have different biological effects. Three major growth factors, vascular endothelial growth factor- (VEGF)-A, VEGF-C and VEGF-D, are involved in the activation process via their receptors (VEGFRs). The purpose of current study is to investigate the significant difference between intratumoral and peritumoral lymphatic vessel density (LVD) in gastric cancer and their correlations with lymphangiogenetic growth factors.

**Methods:**

Intratumoral LVD (I-LVD) and peritumoral LVD (P-LVD) of 123 patients with primary gastric cancer were assessed after staining with D2-40, and confirmed by double staining with D2-40/CD34. Proliferative activity of lymphatics endothelium was evaluated by double staining with D2-40/Ki-67. The associations were analyzed between I-LVD/P-LVD and the expression level of VEGF-A, VEGF-C, VEGF-D and the receptor VEGFR-3, which was measured by immunohistochemistry (IHC). The correlations of I-LVD and P-LVD with patient prognosis were also valued.

**Results:**

(1) The peritumoral lymphatics (PTLs) were relatively enlarged with dilated lumen compared with the intratumoral lymphatics (ITLs). Increased P-LVD was significantly higher than I-LVD (*P *< 0.05). (2) P-LVD was found significantly associated with lymph node metastasis (LNM) (*P *< 0.001), lymphatic vessel invasion (LVI) (*P *< 0.001), VEGF-C (*P *= 0.003), VEGF-D expression level (*P *= 0.005) and VEGFR-3 expression level (*P *< 0.001) in peritumoral tissues, despite no significant association was found between above variants with I-LVD. However, increased I-LVD was demonstrated to be associated with decreased tumor volume (*P *< 0.001). Neither I-LVD nor P-LVD was correlated with VEGF-A expression (*P *> 0.05). (3) Proliferative activity of lymphatics endothelium was observed in PTLs, in spite of ITLs. (4) Increased P-LVD, but not I-LVD, was indicated to be an independent risk factor for lymph node metastasis by multivariate logistic regression analysis, and was related to worse disease-free survival and overall survival.

**Conclusions:**

PTLs play roles in gastric cancer progression. Increased P-LVD, but not I-LVD, was significantly associated with VEGF-C/-D/VEGFR-3 system, and could be an independent risk factor for lymph node metastasis and a prognostic factor in gastric cancer.

## Background

Gastric cancer is the main leading cause of cancer-related death in China. About 80% ~ 90% patients are diagnosed at advanced stage with poor outcome, commonly with lymphatic dissemination and distant metastasis. During the past several years, tumor-induced lymphangiogenesis driven by lymphangiogenic growth factors has been firmly established as a novel mechanism for cancer progression. Nowadays, an increasing number of experts believe that intratumoral lymphatics (ITLs, the lymphtics within the tumors) and peritumoral lymphatics (PTLs, lymphtics at the periphery) play exactly distinct biological roles on tumor behavior and prognosis in different types of tumors. In gastric cancer, several studies have indicated that patients with higher I-LVD had the higher presence of lymph node metastasis in early stage [1], while P-LVD could be an independent risk factor for lymph node metastasis and prognosis [2]. However, function of I-LVD and P-LVD and their correlations with VEGFs expression haven't been clarified yet.

A number of studies have demonstrated the crucial roles of VEGFs expressions on tumor progression and prognosis in gastric cancer. VEGF-C and VEGF-D, two members of VEGF family, have been defined as the lymphangiogenic growth factors and play an important role in tumor lymphangiogenesis via activation of VEGFR-3, which is mainly expressed in lymphatic endothelial cells (LECs). VEGF-C is a dominant regulator of lymphangiogenesis in both early and advanced gastric cancer [3,4]. Increased VEGF-C expression had a significant correlation with LVD, LVI and lymph node metastasis [5], but its prognostic value remained controversial. VEGF-D was involved in lymphatic spreading of gastric cancer cells and could be an independent prognostic marker [6]. VEGFR-3 was also indicated as a prognostic factor [6]. Another growth factor, VEGF-A, which regulated angiogenesis, was also considered to stimulate lymphangiogenesis by binding to VEGFR-2 recently. Increased VEGF-A expression level of gastric cancer patients had been proven to be related with microvessel density (MVD), hematogenous metastasis, peritoneal disseminateion and poor prognosis. However, it remains unknown whether both of the intratumoral and peritumoral lymphtics are stimulated by the three VEGFs secreted by tumor cells, or whether the I-LVD and P-LVD play significantly different biological roles in lymph node metastasis and prognosis in gastric cancer.

## Methods

### Patients and tumor specimens

Tumor specimens were obtained from 123 patients with primary gastric cancer who accepted gastrectomy at Department of Surgery, Tongji Hospital of Tongji University from January 2000 to December 2003. None of them had received preoperational chemotherapy or radiotherapy treatment. The study population consisted of 80 men (65%) and 43 women (35%). The average age at time of diagnosis was 65 years (ranged from 28 to 87 years). Thirty-one cases of early gastric cancer (EGC) and 92 cases of advanced gastric cancer (AGC) were involved in. Histological stage was based on UICC TNM classification. Other clinical features were summarized in Table 1. All patients have been followed up clinically for at least 5 years after surgery. The average follow-up time was 56 months (ranged from 6 to 85 months). Survival analysis was performed, including overall survival (OS), disease-free survival (DFS) and cancer-specific survival (CSS). OS, DFS and CSS was calculated from the date of surgery to last contact for living patients, to the date of the last follow-up for disease-free patients, and to the date of gastric-cancer-induced death, respectively. Four EGC cases and 52 AGC cases were occurred recurrence. Eleven patients had peritoneal dissemination, 26 patients liver metastasis, and 19 cases relapsed in the stomach after operation. Forty patients died of gastric cancer. The current study was approved by the Research Ethics Committee of Tongji Hospital affiliated with Tongji University. The normal gastric tissues were collected as control specimens. All the results were accomplished by two pathologists independently, and the means were calculated for each case based on the data obtained.

**Table 1:** Correlations of LVD with clinicopathological parameters and VEGFs expressions

Factors	N	I-LVD		P-LVD	
			
		mean ± SD	*P*	mean ± SD	*P*
Tumor differentiation			0.802		0.739
High differentiated	11	8.18 ± 2.44		12.07 ± 4.49	
Mederately/poor differentiated	112	8.00 ± 2.27		12.46 ± 3.60	
Tumor size			<0.001		<0.001
<3.2 cm	57	8.93 ± 2.34		11.18 ± 3.80	
≥ 3.2 cm	66	7.23 ± 1.91		13.50 ± 3.21	
Depth of invasion			0.433		0.026
pT1-2	69	8.16 ± 2.51		11.78 ± 3.52	
pT3-4	54	7.83 ± 1.96		13.26 ± 3.71	
Lymph node metastasis			0.056*		<0.001
Negative	57	8.47 ± 2.27		10.50 ± 3.42	
Positive	66	7.65 ± 2.24		13.98 ± 3.10	
LVI			0.700		<0.001
Negative	83	8.08 ± 2.33		11.23 ± 3.29	
Positive	40	7.91 ± 2.22		14.36 ± 3.44	
VI			0.905		<0.001
Negative	86	8.00 ± 2.38		11.65 ± 3.81	
Positive	37	8.05 ± 2.67		14.24 ± 2.53	
TNM stage			0.067		<0.001
I - II	71	8.34 ± 2.42		11.33 ± 3.29	
III - IV	52	7.58 ± 2.01		13.92 ± 3.66	
VEGF-A expression			0.527		0.091
Low	44	7.84 ± 2.51		11.68 ± 3.54	
High	79	8.26 ± 2.21		12.84 ± 3.69	
VEGF-C expression			0.092*		0.003
Low	42	7.55 ± 2.37		11.06 ± 3.26	
High	81	8.00 ± 2.34		13.13 ± 3.69	
VEGF-D expression			0.514		0.005
Low	72	7.90 ± 2.47		11.65 ± 3.44	
High	51	8.18 ± 1.99		13.53 ± 3.73	

*P*, t test; P*, Mann-Whitney *U *Test. Abbreviation: LVD: lymphatic vessel density; LVI: lymphatic vessel invasion. VI: venous invasion.

### Single immunohistochemistry for D2-40, VEGF-A, VEGF-C, VEGF-D and VEGFR-3

For immunohistichemical staining, 4 μm-thick paraffin-embedded slides were cut from each study block. Sections were treated with 0.3% H_2_O_2 _for 10 min at room temperature. For antigen retrieval, the slides were heated in a microwave oven containing 0.01 mmol/L sodium citrate (pH 6.0). Slides were incubated at 4°C overnight in a humidity tray with primary antibodies, VEGF-A (mouse monoclonal antibody, 1:100, DAKO), VEGF-C (goat polyclonal antibody, 1:100, Santa Cruz Biotechnology, Santa Cruz, CA), VEGF-D (goat polyclonal antibody, 1:100, Santa Cruz), VEGFR-3 (goat polyclonal antibody, 1:100, Santa Cruz) and D2-40 (mouse monoclonal antibody, 1:100, DAKO), respectively. Slides were rinsed thrice in 0.1 mmol/L PBS for 2 min, and incubated for 30 min at room temperature with goat anti-rabbit/mouse horseradish peroxidase (Envision, DAKO, CA) to identify the target. The sections were developed with 3' 3-diaminobenzidine. The normal goat IgG was served as negative reaction control for staining of VEGF-C, VEGF-D and VEGFR-3, and the normal rabbit IgG was served as negative reaction control for staining of VEGF-A and D2-40.

### Double immunohistochemical staining for D2-40/CD34

The double immunohistochemical staining for D2-40/CD34 was further processed to evaluate the specificity of D2-40 expression in lymphatic endothelium. The proposal was according to the manufacturer's instruction (No: 95-9999, Histostain-DS Kit, Zymed, CA). Paraffin-embedded 4 μm sections were deparaffinized with xylene and rehydrated. The slides were submerged in peroxidase quenching solution for 10 min. After being incubated overnight at 4°C with the primary antibodies against CD34 (mouse monoclonal antibody, 1:100, DAKO, Carpinteria, CA), the sections were treated with serum blocking solution, followed by being incubated with the biotinylated secondary antibody (DAKO). Subsequently, the alkaline phosphatase conjugate was used to each section for 10 min. And then, the sections were treated with the substrate chromogen mixture and double staining enhancer. The serum blocking solution was applied again, the slides were incubated with another primary antibody against D2-40 (mouse monoclonal antibody, 1:200, GM36190, Gene Tech Company Limited, Shanghai, China) for 60 min and the biotinylated second anti-immunoglobulin (Ig) (DAKO) was treated. After applying with enzyme conjugate for 10 min, the slides were incubated with the mixture of substrate buffer, chromogen solution and 0.6% hydrogen peroxide for HRP and monitored under a microscope. Tap water containing 0.05% Tween-20 terminated the reaction. For negative controls, the sections were stained with a non-immune serum instead of the same concentration of primary antibody. The CD34 positive blood vessels showed the intense red stain, and the D2-40 positive lymphatic vessels showed the dark purple stain.

### Double staining for D2-40/Ki-67

For detecting the proliferative activity of the lymphatic vessels, the method of double immunostaining for D2-40/Ki-67 was performed. The D2-40 antibody was used to stain lymphatic vessels endothelium, together with Ki-67 (rabbit monoclonal antibody, 1:200, Santa Cruz, USA) to stain proliferative cells. Ki-67 staining (red) was developed with alkaline phosphatase-conjugated secondary antibody, and then D2-40 staining (brown) was developed with peroxidase-conjugated secondary antibody. Proliferative lymphatic vessels were confirmed by Ki-67 positively stained nuclei that also had concomitant positive cytoplasmic staining in D2-40 positive cells. The rate of double-labeled vessels was determined by counting the nuclei of tumor-associated D2-40-positive vessels (100 nuclei in each tumor) [7].

### Assesment of LVD

Intratumoral LVD (hot spots were located at tumor center) and peritumoral LVD (hot spots were located at periphery tissue within 2 mm of tumor adjacent to the invasive front) were assessed separately [2,7-9]. Quantitative analysis of lymphatic vessel density was performed in sections which were single-stained for D2-40. Five areas with most lymphatics regions ("hot spots") were chosen at × 40 magnification by light microscopy. LVD was assessed by counting all stained vessels at × 200 magnification. The mean number of lymphatics assessed was determined as LVD. Lymphatic vessel invasion (LVI) was detected to be present if at least one tumor cell cluster was in D2-40 positive vessels [10]. Scoring and counting were performed independently by two investigators who had no clinical information of the patients. The mean P-LVD and I-LVD were calculated for each case.

### Assesment of VEGFs and VEGFR-3 expressions

Positive staining of VEGF-A, -C and -D expression was defined as the previous studies [6,11]. Staining results for the above three VEGFs were semiquantitatively assessed by an immunohistochemical score combined with the percentage of tumor cells showing specific immunoreactivity. Staining intensity was given with four grades: none (0), weak (1), moderate (2) and strong (3). The percentage of positive carcinoma cells was given with the grades: 0 (0%), 1 (1% ~ 10%), 2 (11% ~ 49%), 3 (50% ~ 100%), respectively. The total score was calculated by multiplying the staining intensity and the percentage of positive tumor cells. The median of the score was selected as the cutoff level according to which tumors were categorised into low- (0 ~ 3) and high-expression (4 ~ 6) tumors [6,11]. VEGFR-3-positive vessels were determined as described earlier [12]. The vessels of three hot spots areas were counted at × 400 magnification. Staining was considered as positive when more than 5% of endothelium showed a staining [6]. Peritumoral VEGFR-3-positive vessels (P-VEGFR-3) and intratumoral VEGFR-3-positive vessels (I-VEGFR-3) were assessed as above LVD.

### Statistical analysis

Statistical analysis was performed using the Statistics Package for the Social Science software (version 11.5; SPSS Inc, Chicago, IL). Specimens were divided into two categories according to the median values of I-LVD and P-LVD, respectively. The correlations of I-LVD and P-LVD with clinicopathologic parameters and VEGFs expressions were analysed by independent samples *t *test or Mann-Whitney *U *test. The correlations of VEGFR-3 expression with clinical parameters were analysed by Pearson Chi-Square Tests. The related factors about lymph node metastasis in gastric cancer were accomplished using the multivariant logistic regression analysis. Survival curves were obtained using Kalplan-Meier method and compared by log-rank test. Multivariate survival analysis was evaluated using Cox's proportional hazard method. All the statistical analysis was two sides with significance defined as *P *< 0.05.

## Results

### Intratumoral and peritumoral lymphatic characteristics in gastric cancer

**Figure 1:**
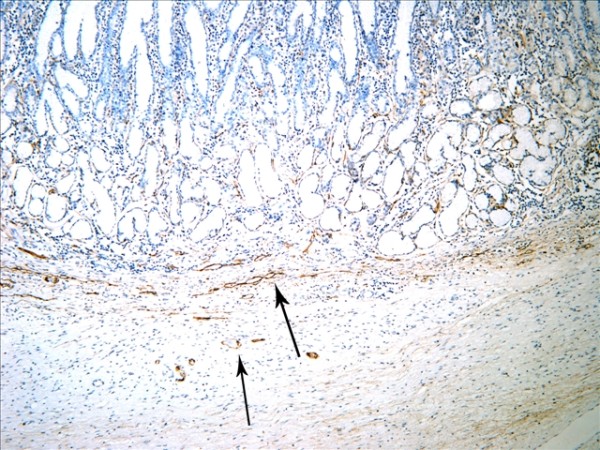
**The D2-40-positive lymphantics mainly located at the layer of submucosa in gastric tissue (arrows). **IHC, magnification: ×200.

**Figure 2:**
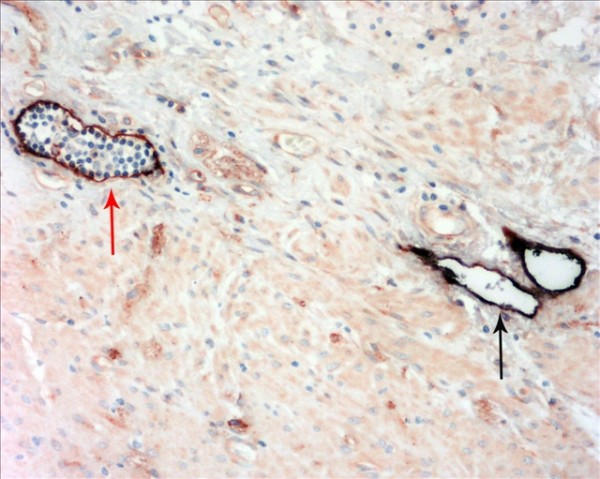
**The double immunohistochemical staining for D2-40 and CD34 clearly distinguished the lymphatic vessels (black arrow) from blood vessels (red arrow)**. IHC, magnification: ×400.

**Figure 3:**
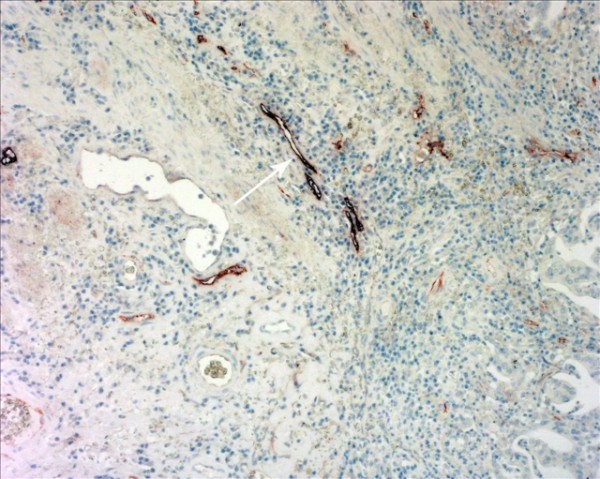
**The intratumoral lymphatic vessels in gastric cancer were collapsed (arrow); Double immunohistochemical staining**, magnification: ×200.

**Figure 4:**
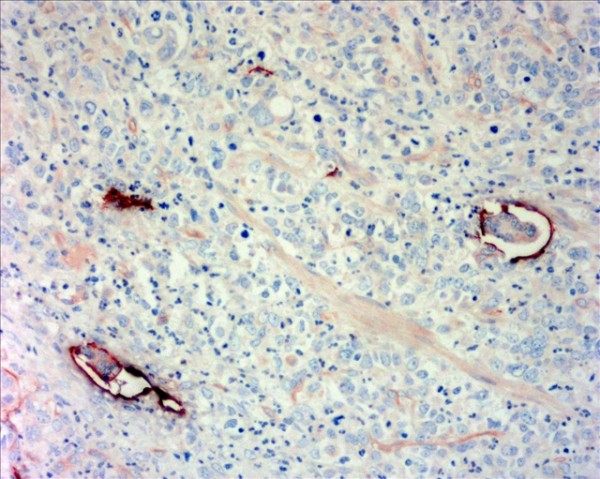
**The invading cancer cells cluster were present in lymphatic vessel invasion (LVI)**. Double immunohistochemical staining for D2-40/CD34, magnification: ×400.

**Figure 5:**
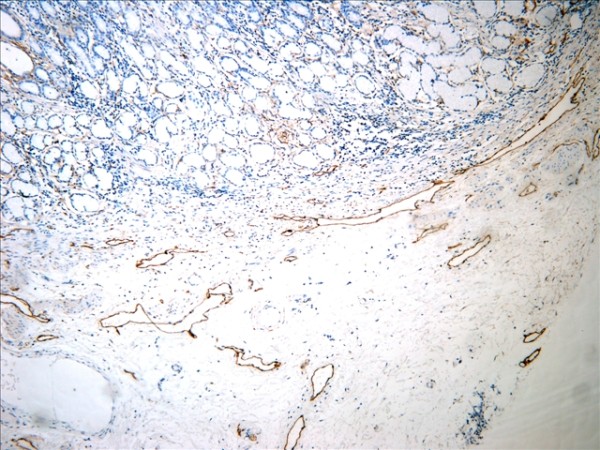
**The peritumoral lymphatic vessels in gastric cancer were enlarged with dilated lumen located at the superficial submucosa**. IHC, magnification: ×200.

**Figure 6:**
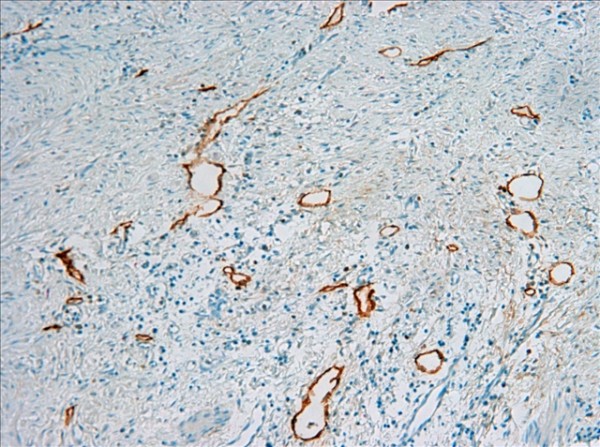
**The peritumoral lymphatic vessels in gastric cancer were enlarged with dilated lumen located at deep part of submucosa**. IHC, magnification: ×200.

The D2-40-positive lymphatic vessels had irregular morphology and thin-walled lumen. Lymphatic vessels in gastric tissues were mostly located in the layer of submucosa (Figure 1). The lymphatic vessels (D2-40-position) and blood vessels (CD34-position) were clearly distinguished by further double staining (Figure 2). Intratumoral lymphatic vessels usually were collapsed, small and irregular (Figure 3), but some noncollapsed lymphatics showed open lumen, and occasionally contained invading tumor cells clusters (Figure 4). The peritumoral lymphatic vessels in the superficial and deep part of submucosa were all enlarged with the lymphatic cavities dilated (Figure 5, Figure 6). The lymphatic vessel invasion was observed in 38 cases. No significant correlation was found between the numbers of LVD in tumor center and in control tissues (8.02 ± 2.28 vs 8.13 ± 1.04, *P *> 0.05). However, the numbers of P-LVD (12.15 ± 3.75) were significantly higher than that in control tissues and tumor center (*P *< 0.05). No statistical differences was found between the two methods of detecting lymphatics (single staining for D2-40 and double staining for D2-40/CD34) (I-LVD, 8.02 ± 2.28 vs 7.80 ± 2.33; P-LVD, 12.15 ± 3.75 vs 12.42 ± 3.67, *P *> 0.05).

### Correlations of I-LVD and P-LVD with clinicopathologic parameters and VEGFs expressions

The correlations of I-LVD and P-LVD with clinicopathologic parameters were shown in Table 1. Increased I-LVD was significantly associated with smaller tumor size (*P *< 0.001). No correlations was found between I-LVD and VEGFs expressions, while P-LVD was significantly correlated with larger tumor size (*P *< 0.001), depth of invasion (*P *= 0.026), lymph node metastasis (*P *< 0.001), LVI (*P *< 0.001), venous invasion (VI) (*P *< 0.001), TNM stage (*P *< 0.001), VEGF-C (*P *= 0.003) and VEGF-D expression (*P *= 0.005).

**Figure 7:**
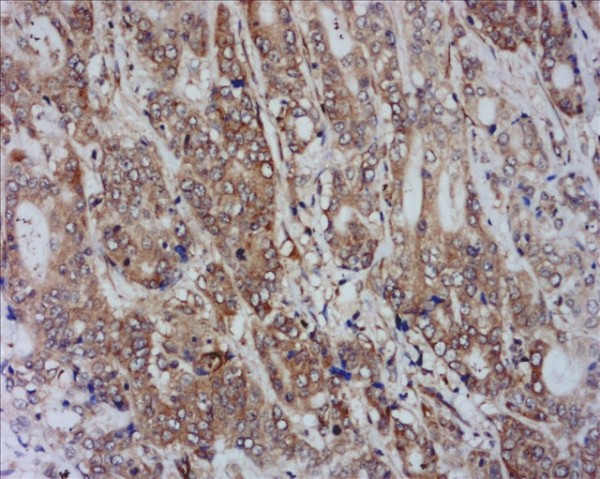
**VEGF-A expression in the cytoplasm in gastric cancer**. IHC, magnification: ×400.

**Figure 8:**
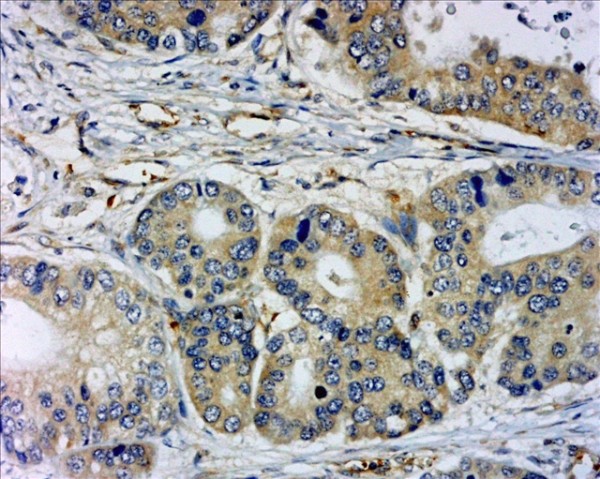
**VEGF-C expression in the cytoplasm in gastric cancer**. IHC, magnification: ×400.

**Figure 9:**
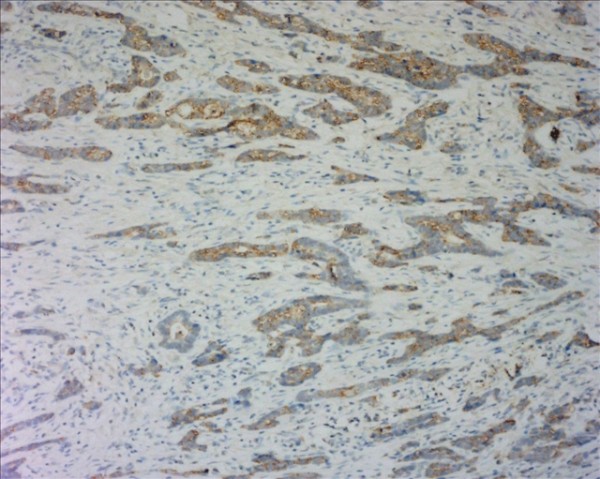
**VEGF-D expression in the cytoplasm in gastric cancer**. IHC, magnification: ×200.

Expression of the three VEGFs showed a positive cytoplasmic staining in gastric cancer cells. High expression level of VEGF-A (Figure 7), VEGF-C (Figure 8) and VEGF-D (Figure 9) were observed in 64.2% (79/123), 65.9% (81/123) and 41.5% (51/123) of specimens, respectively. Neither I-LVD nor P-LVD was found significantly associated with VEGF-A expression (*P *> 0.05).

### Proliferative activities in intra- and peritumoral lymphatics

**Figure 10:**
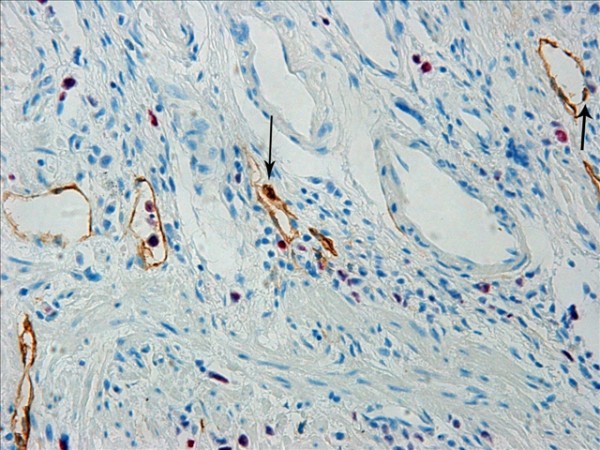
**Ki-67-positive lymphatic vessel nuclei (arrows) were detected in the tumor periphery**. Double staining for D2-40/Ki-67, magnification: ×400.

**Figure 11:**
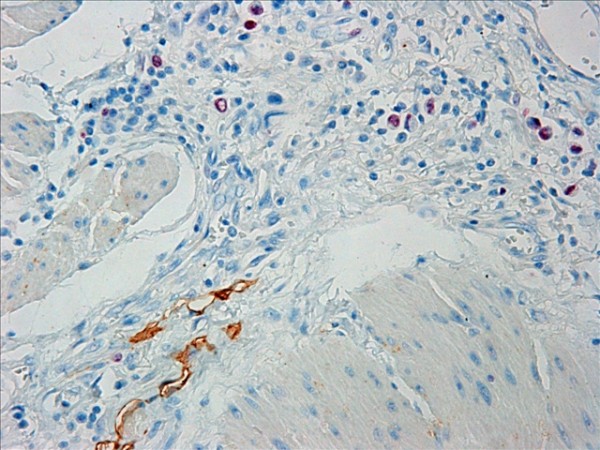
**No Ki-67 positive expression in the intratumoral lymphatics nuclei**. Double staining for D2-40/Ki-67, magnification: ×400.

**Figure 12:**
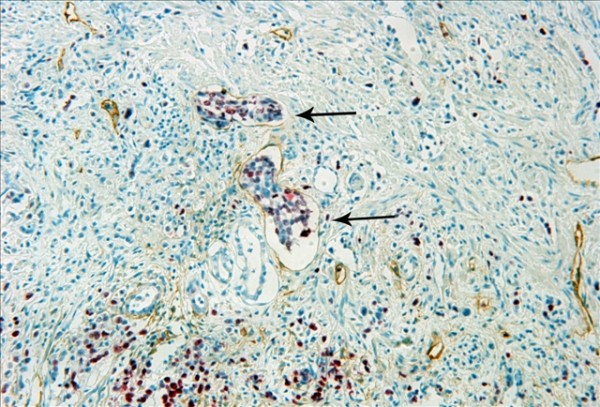
**Lymphatic vessels invasion was detected in the peritumoral tissue (arrows)**. Double staining for D2-40/Ki-67, magnification: ×400.

Proliferative lymphatic vessels were found in the tumor periphery, (Figure 10). The rate of Ki-67-positive lymphatic vessel nuclei in the tumor periphery was (0.81 ± 0.13)%. No proliferative lymphatic vessel was found in the tumor center (Figure 11). Lymphatics invasion could be observed in the peritumoral tissues (Figure 12).

### VEGFR-3 expression in lymphatic vessels

**Figure 13:**
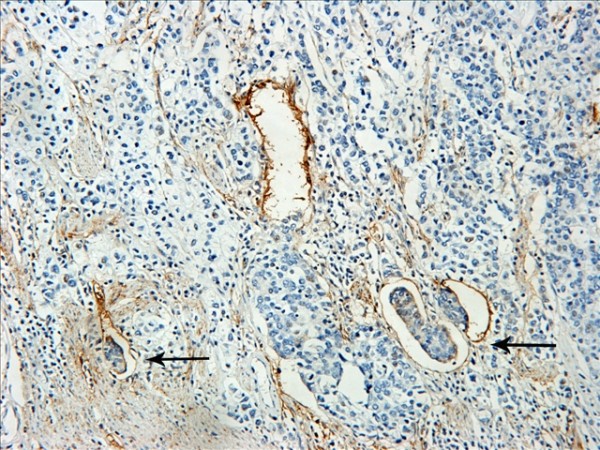
**The VEGFR-3-positive expression in the endothelial cells cytoplasm in tumor periphery (P-VEGFR-3), occasionally with cancer cell clusters invading (arrows)**. IHC, magnification: ×400.

**Figure 14:**
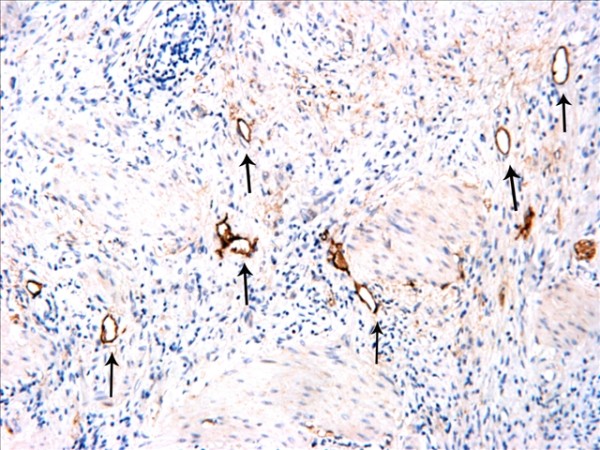
**The VEGFR-3-positive expressions were located at tumor center (arrows)**. IHC, magnification: ×200.

The VEGFR-3-positive expression in tumor periphery (P-VEGFR-3) was found in 55 of the 123 cases, occasionally with cancer cell clusters invading (Figure 13). However, only 34 of 123 cases had a VEGFR-3-positive expression at the tumor center (I-VEGFR-3) (Figure 14). These vessels were mostly thin-walled, irregular shaped and contained no or few RBCs.

In peripheral tumor tissues, VEGFR-3 expressions were significantly correlated rather with VEGF-C high expression (*P *< 0.000), VEGF-D high expression (*P *= 0.043), increased P-LVD (*P *< 0.001) and the presence of LVI (*P *= 0.003), than with VEGF-A expression, increased I-LVD, venous invasion and lymph node metastasis (*P *> 0.05). No correlations were observed between I-VEGFR-3 expression and clinical parameters (*P *> 0.05). (Table 2)

**Table 2:** Correlations of VEGFR-3 expression at differenr tumor location with clinical parameters

Factors	N	I-VEGFR-3		P-VEGFR-3	
			
		N (%)	*P*	N (%)	*P*
VEGF-A expression			0.107		0.067
Low	44	16(36.36)		15(34.09)	
High	79	18(22.78)		40(50.63)	
VEGF-C expression			0.795		< 0.001
Low	42	11(26.19)		9 (21.43)	
High	81	23(28.40)		46(56.79)	
VEGF-D expression			0.968		0.043
Low	72	20(27.78)		27(37.50)	
High	51	14(27.45)		28(54.90)	
P-LVD			0.813		<0.001
<14	60	16(26.67)		10(16.67)	
≥ 14	63	18(28.57)		45(71.43)	
I-LVD			0.412		0.153
<8	58	14(24.14)		22(37.93)	
≥ 8	65	20(30.77)		33(50.77)	
Lymph node metastasis			0.934		0.190
Negative	55	19(34.55)		21(38.18)	
Positive	68	15(22.06)		34(50.00)	
LVI			0.681		0.003
Negative	76	22(28.95)		26(34.21)	
Positive	47	12(25.53)		29(61.70)	
VI			0.387		0.448
Negative	87	26(29.89)		37(42.53)	
Positive	36	8 (22.22)		18(50.00)	

Abbreviation: LVD: lymphatic vessel density; LVI: lymphatic vessel invasion; VI: venous invasion; I-VEGFR-3: VEGFR-3 positive expression in the tumor center; P-VEGFR-3: VEGFR-3 positive expression at the tumor periphery.

### Predictive value of LVD for lymph node metastasis

As a result of multivariate logistic regression analysis in Table 3, VEGF-C expression and P-LVD were significantly asscoiated with lymph node metastasis (*P *= 0.024, *P *= 0.045, respectively). I-LVD, VEGF-A, VEGF-D and P-VEGFR-3 expression did not show the predictive value for lymph node metastasis in gastric cancer. (Table 3)

**Table 3:** Multivariate logistic regression analysis for lymph node metastasis

Factors	Odds ratio	95% CI	*P*
P-LVD	3.548	1.030 -12.226	0.045
I-LVD	1.300	0.964 - 1.754	0.085
VEGF-A	0.510	0.138 - 1.884	0.313
VEGF-C	4.069	1.198 - 13.820	0.024
VEGF-D	3.162	0.834 - 11.992	0.091
P-VEGFR-3	2.919	0.747 - 11.403	0.123

Abbreviation: LVD: lymphatic vessel density; P-VEGFR-3: VEGFR-3 positive expression at the tumor periphery.

### Prognostic significance of I-LVD and P-LVD

**Figure 15:**
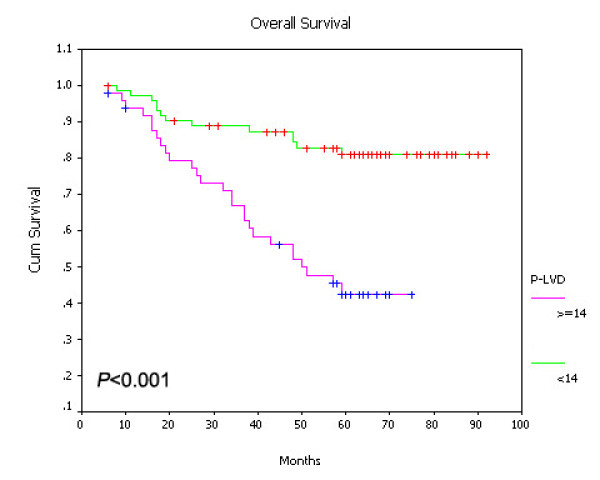
**Relationship between P-LVD with overall survival (*P *< 0.001)**.

**Figure 16:**
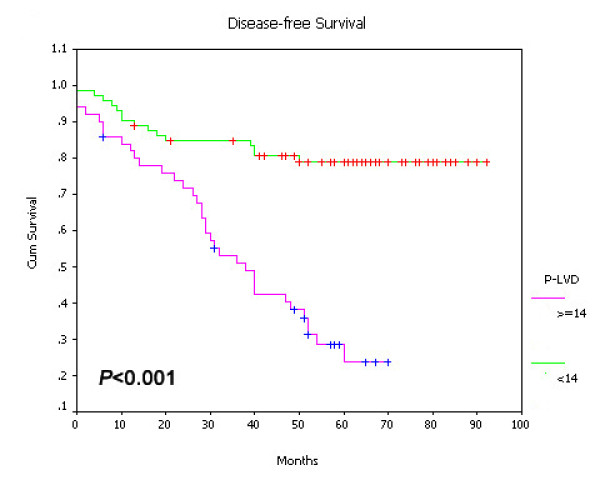
**Relationship between P-LVD with disease-free survival (*P *< 0.001)**.

**Figure 17:**
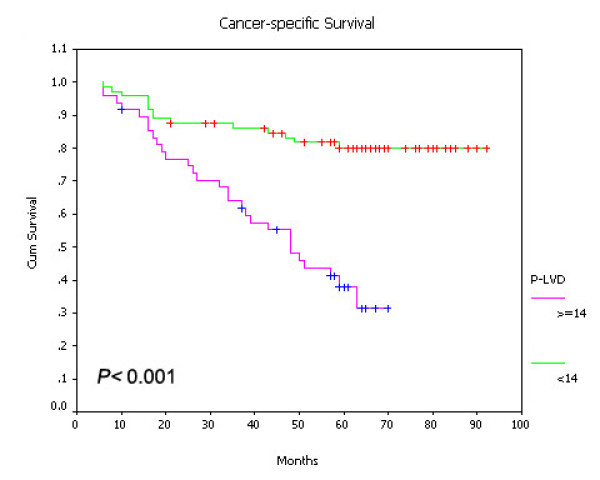
**Relationship between P-LVD with cancer-specific survival (*P *< 0.001)**.

**Figure 18:**
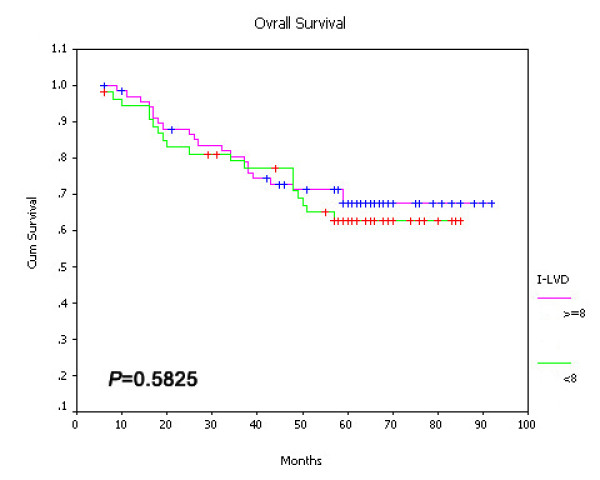
**Relationship between I-LVD with overall survival (*P *= 0.5825)**.

**Figure 19:**
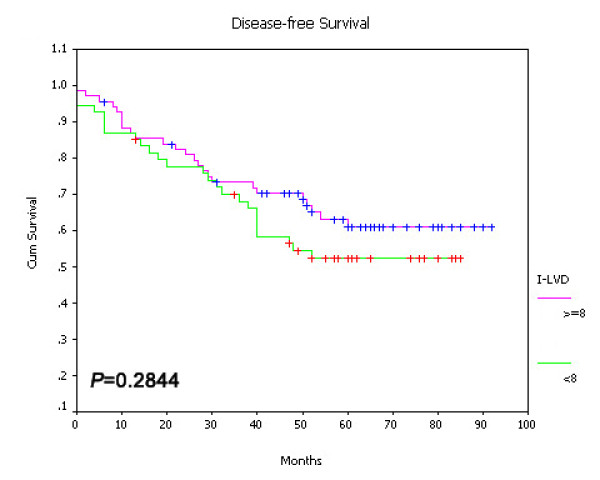
**Relationship between I-LVD with disease-free survival (*P *= 0.2844)**.

**Figure 20:**
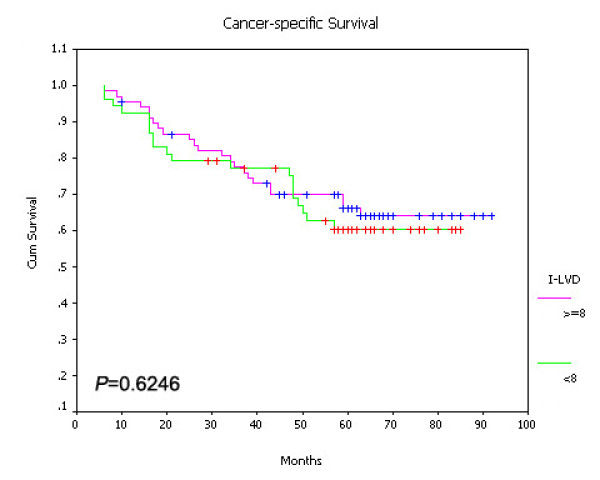
**Relationship between I-LVD with cancer-specific survival (*P *= 0.6246)**.

On univariate survival analysis, P-LVD was associated with poor overall survival (Figure 15, *P *< 0.001), disease-free survival (Figure 16, *P *< 0.001) and cancer-specific survival (Figure 17, *P *< 0.001). However, I-LVD was correlated with a nonsignificantly trend towards all the above respectively (overall survival, *P *= 0.5835, Figure 18; disease-free survival, *P *= 0.2844, Figure 19; cancer-specific survival, *P *= 0.6246, Figure 20).

Multivariate regression analysis indicateded that P-LVD could be an independent prognostic factor for both overall survival (*P *= 0.045) and disease-free survival (*P *= 0.031), despite cancer-specific survival. Moreover, presence of LVI and TNM stage could serve as the independent predictors for all the three survivals (LVI, *P *= 0.040, 0.043, 0.039; TNM stage, *P *= 0.048, 0.001, 0.001, Table 4). VEGF-A expression was the independent prognostic predictor only for overall survival (*P *= 0.033). No statistically significant correlations for I-LVD, VEGF-C, VEGF-D and P-VEGFR-3 with any of the survivals were found. (Table 4)

**Table 4:** Cox regression analysis of independent factors affecting disease-free survival, cancer-specific survival and overall survival

Factors	Cancer-Specific Survival		Disease-Free Survival		Overall Survival	
			
	OR (95% CI)	*P*	OR (95% CI)	*P*	OR (95% CI)	*P*
P-LVD	2.099(0.91-4.87)	0.084	2.418(1.08-5.40)	0.031	2.895 (1.02-8.19)	0.045
I-LVD	1.205(0.56-2.63)	0.639	1.325(0.62-2.85)	0.472	1.959 (0.90-4.24)	0.088
VEGF-A	1.386(0.50-3.83)	0.528	1.364(0.67-2.78)	0.391	2.437(1.10-5.45)	0.030
VEGF-C	2.423(0.89-6.63)	0.085	1.630(0.77-3.47)	0.205	1.562 (0.79-3.34)	0.182
VEGF-D	0.511(0.19-1.36)	0.178	1.916(0.90-4.10)	0.094	1.621 (0.81-3.98)	0.190
LVI	2.716 (1.05-7.04)	0.040	2.477(1.03-5.97)	0.043	2.578 (1.05-6.34)	0.039
LNM	2.115(0.68-6.60)	0.197	2.428(1.18-5.02)	0.017	3.426 (1.66-7.06)	0.001
VI	2.943(0.89-9.77)	0.078	1.697(0.75-3.87)	0.208	1.477(0.63-3.48)	0.373
P-VEGFR-3	1. 499(0.98-2.31)	0.067	1.099(0.53-2.28)	0.800	1.402 (0.68-2.90)	0.361
TNM stage	1.523(1.00-2.31)	0.048	1.674(1.25-2.25)	0.001	1.656(1.23-2.23)	0.001

Abbreviation: P-LVD: peritumoral lymphatic vessel density; I-LVD: intratumoral lymphatic vessel density; LVI: lymphatic vessel invasion; LNM: lymph node metastasis; VI: venous invasion; P-VEGFR-3: VEGFR-3 positive expression at the tumor periphery.

## Discussion

Recently, the D2-40 antibody, a new marker for lymphatic endothelium, was identified as the specific antibody against human podoplanin [13]. Many studies have indicated the immunostaining of D2-40 is specific for evaluation of lymphatic invasion and lymphatic microvessel density in human cancers, including in gastric cancer [14-17]. In this study, LVD and lymphatic vessel invasion were identified by D2-40 staining, and confirmed by double staining for D2-40 and CD34, which clearly discriminated lymphatics from blood vessels further. Our results also showed the numbers of lymphatic vessel density evaluated by the two methods had no statistical significance. That meant the D2-40 was specific for lymphatic endothelium in gastric cancer. Similar to the precious reports, the D2-40-positive lymphatic vessels in our study usually had the irregular shape and thin-walled lumen containing no RBCs.

The different morphology of ITLs and PTLs was observed here. The ITLs mostly were collapsed, small and had less numbers than PTLs, which had obviously increased numbers with dilated lumen. The larger tumor size was, the less the numbers of I-LVD were. This result was in agreement with other report in advanced gastric cancer [2]. Its reason was postulated as that the lymphatics in the tumor tissue were destroyed by invaded tumor cells, or high interstitial fluid pressure caused by expanding tumor cell masses and growing malignant cells in a confined space [18]. Although some noncollapsed intratumoral lymphatics had open lumen and occasionally contained invading tumor cells, our results didn't show the linkage of I-LVD with lymphatic invasion and lymph node metastasis. However, Peng Gao *et al*. indicated I-LVD was correlated with LVI and lymph node metastasis in early gastric cancer [2]. In this study, while despite that I-LVD had a trend of correlation with LNM, no statistical significance was found (*P *= 0.056). Except for the above, few studies about I-LVD in gastric cancer were reported.

The significant association of ITLs with LNM was observed in other human tumors, paplillary thyroid carcinoma [19], for instant. The increased I-LVD was correlated with neck node metastasis in oropharyngeal carcinoma [20] and associated with lymphatic invasion in pancreatic endocrine tumors [21]. Wherever the lymphangiogenesis occurs in peritumoral and intratumoral tissues, the association of it with regional LNM is mainly dependent on whether the new-formed lymphatics are functional, or not [18]. The function of LECs was measured by Ki-67 expression in the nuclei, however, we didn't observe the proliferative activity in ITLs endothelium. Furthermore, correlation wasn't obseved between I-LVD and VEGF-A, VEGF-C, VEGF-D and VEGFR-3 expression level in the intratumoral tissues. Therefore, ITLs in gastric cancer were unfunctional according to our results. Although tumor-secreted VEGF-C/-D and, even VEGF-A to a lesser extent, are important for inducing intratumoral lymphangiogenesis in solid tumor mass [22], our results demontrated that ITLs in gastric cancer didn't be stimulated by VEGF-C/-D-mediated VEGFR-3 signaling pathway or VEGF-A expression. Similar to our observations, no relationships were found between ITLs proliferation and VEGF-C/VEGFR-3 expression in head and neck cancer and cutaneous melanoma [9,20]. However, I-LVD was correlated with VEGF-C expression in pancreatic endocrine tumors [21].

In contrast to ITLs and I-LVD in the present study, the PTLs endothelium showed the proliferative activity with Ki-67 expression on D2-40-positive lymphatics, and increased P-LVD was correlated with LVI and LNM. Our results demonstrated that PTLs were functional in gastric cancer, and the dilated PTLs increased drainage activity in the tumor periphery. Furthermore, positive correlations were observed between P-LVD and VEGF-C, VEGF-D and VEGFR-3 expression in the tumor periphery (P-VEGFR-3), which meant VEGF-C and VEGF-D induced the peritumoral lymphangiogenesis via stimulating VEGFR-3 expression on LECs. Our observation also showed the closely relationships between P-VEGFR-3 and VEGF-C/VEGF-D expression. The increased number of functional and draining PTLs with dilated lumen may collect more metastatic cancer cells from the tumor surface, and then facilitate lymphatics invasion and metastasis [23]. Our results were in agreement with the findings in cutaneous melanoma, squamous cell carcinomas of the head and neck, and gastric cancer investigated by other authors [1,5,9,10,24-26]. VEGF-A expression was not correlated with P-LVD and I-LVD in this study. Even demonstrated in xenograft fibrosarcomas, VEGF-A has induced PTL growth in avascular cormea and promoted lymph node metastasis via VEGF-C/-D/VEGFR-3-independent pathway [27], its role on lymphangiogenesis remains undetermined in human tumors. In gastric cancer, VEGF-A and VEGF-C may play the distinct role: VEGF-A is more likely to be associated with haematogenous metastasis, while VEGF-C is indictive of lymphatic metastasis [28].Other report showed the increased expression of VEGF-A as well as VEGF-C expression is essential in lymph node metastasis [29].VEGF-A stimulates the tumor angiogenesis through activation of VEGFR-1 and VEGFR-2. However, its mechanism of inducing lymphangiogenesis and the function of lymphantics it induced need further studied.

In early gastric cancer, P-LVD and I-LVD could be risk factors for lymph node metastasis [1]. Our results indicated that only P-LVD was the predictor for lymph node metastasis in gastric cancer, but not I-LVD. Additionally, increased P-LVD was associated with worse disease-free survival and overall survival, but not with cancer-specific survival. No correlations were found between I-LVD and patients' prognosis in this study. Multivariate analysis indicated increased P-LVD could be the prognostic factors for disease-free survival and overall survival in gastric cancer, and as an independent predictor for lymph node metastasis. Our results were similar with some other studies. Increased peritumoral lymphangiogenesis may be an indicator of risk of LNM in patients with head and neck squamous cell carcinoma [26]. However, in cutaneous melanoma, increased P-LVD was significantly correlated with improved patient survival, while decreased P-LVD became the predictor of poor prognosis [9]. Since most solid tumors metastasize via lymphatic invasion, LNM becomes an important prognostic factor for the patients' outcome [18], our results also showed that LNM and LVI were the prognostic factors for disease-free survival and overall survival in gastric cancer, and LVI was associated with cancer-specific survival further. Only VEGF-A expression had the significant correlation with overall survival. VEGF-C was another independent risk factor for LNM. The roles of P-LVD and I-LVD in human tumors exist differently. It may be due to the various biological behaviors of tumor cells in different types of human tumors, or the differences in patient selection, experimental design and analytic method.

## Conclusions

Our results suggested PTLs, rather than ITLs, are functional in gastric cancer. Increased P-LVD has the correlation with VEGF-C/-D/VEGFR-3 system, and could be as the independent risk factor for lymph node metastasis and prognostic factor in gastric cancer.

## Competing interests

The authors declare that they have no competing interests.

## Authors' contributions

XW was the guarantor of integrity of the entire study, designed the research, analyzed the data and drafted the manuscript; JF and RT participated in the experiments of immunohistochemical and double immunohistochemical staining. XC guided most of the works. All authors have read and approved the final manuscript.

## Pre-publication history

The pre-publication history for this paper can be accessed here:

http://www.biomedcentral.com/1471-2407/10/299/prepub
